# Blockchain-Based Healthcare Workflow for Tele-Medical Laboratory in Federated Hospital IoT Clouds

**DOI:** 10.3390/s20092590

**Published:** 2020-05-02

**Authors:** Antonio Celesti, Armando Ruggeri, Maria Fazio, Antonino Galletta, Massimo Villari, Agata Romano

**Affiliations:** 1 Department of MIFT, University of Messina, 98122 Messina, Italy; armruggeri@unime.it (A.R.); mfazio@unime.it (M.F.); angalletta@unime.it (A.G.); mvillari@unime.it (M.V.); 2 INdAM-GNCS, 00185 Rome, Italy; 3 IRCCS Centro Neurolesi Bonino Pulejo, 98124 Messina, Italy; 4 ASP Messina, 98123 Messina, Italy; romano.agata16@gmail.com

**Keywords:** blockchain, smart contract, workflow, healthcare, hospital, cloud, IoT, federation

## Abstract

In a pandemic situation such as that we are living at the time of writing of this paper due to the Covid-19 virus, the need of tele-healthcare service becomes dramatically fundamental to reduce the movement of patients, thence reducing the risk of infection. Leveraging the recent Cloud computing and Internet of Things (IoT) technologies, this paper aims at proposing a tele-medical laboratory service where clinical exams are performed on patients directly in a hospital by technicians through IoT medical devices and results are automatically sent via the hospital Cloud to doctors of federated hospitals for validation and/or consultation. In particular, we discuss a distributed scenario where nurses, technicians and medical doctors belonging to different hospitals cooperate through their federated hospital Clouds to form a virtual health team able to carry out a healthcare workflow in secure fashion leveraging the intrinsic security features of the Blockchain technology. In particular, both public and hybrid Blockchain scenarios are discussed and assessed using the Ethereum platform.

## 1. Introduction

Recent advancements in Information and Communication Technology (ICT) have paved the way toward new innovative tele-healthcare services able to face the growing demand of even more accessible medical treatments [1,2]. Moreover, in the pandemic condition such as that we are living at the time of writing of this paper due to the Covid-19 virus, the need of tele-healthcare service becomes dramatically fundamental to reduce the movement of patients, thence reducing the risk of infection. However, the recent innovation brought by Cloud computing and Internet of Things (IoT) paradigms have been only partially taken into consideration by hospitals and more in general by medical centers so far. In this regard, a crucial aspect that has slowed down the wide adoption of such ICT paradigms in hospitals has regarded integrity, security and privacy of exchanged data. Considering the healthcare domain, it is fundamental that shared pieces of clinical data must be certified and not corrupted to prevent intentional or accidental illegal data manipulation. Furthermore, patients’ privacy must be guaranteed.

In recent years, Cloud computing and IoT paradigms along with the concept of the federation have been combined so that different variants born. The first paradigm variation regarded Cloud federation that was defined as a mesh of Cloud providers that are interconnected to provide a universal decentralized computing environment where everything is driven by constraints and agreements in a ubiquitous, multi-provider infrastructure [3]. With the advent of IoT, the IoT Cloud paradigm raised. It was defined as a distributed system consisting of a set of smart embedded devices interconnected with a remote Cloud infrastructure, platform or software through the Internet able to provide IoT as a Service (IoTaaS). Furthermore, the natural evolution of the latter brought to the concept of IoT Cloud federation referred as an ecosystem composed of small, medium and large IoT Cloud providers able to federate themselves to gain economies of scale and to enlarge their processing, storage, network, sensing and actuating capabilities to arrange more flexible IoTaaS [4]. The healthcare domain can benefit from these paradigms to improve clinical services and push down management costs through the creation of Hospital IoT Clouds [5] able to federate themselves.

In this paper, we focus on medical laboratory as a case study. It is an applied science laboratory typically placed in a hospital or in a clinical centre where clinical pathology exams are carried out on clinical samples to obtain information about the health of a patient to make diagnosis, treatment and prevention of diseases. Blood tests (e.g., complete blood count (CBC), glycaemia and so on) are performed by biomedical laboratory health technicians directly in the clinical laboratory and results are validated and analyzed by doctors to make therapies. Specifically, leveraging the IoT Cloud federation paradigm we propose the emerging concept of tele-medical laboratory. It is a medical laboratory where clinical exams are performed on patients directly in a hospital by technicians through IoT medical devices interconnected with a Hospital Cloud system and results are automatically sent through the hospital Cloud to doctors of federated hospitals for validation and/or consultation. Biomedical laboratory health technicians, nurses, doctors and other clinical personnel belonging to different Federated Hospital IoT Clouds (FHCs) cooperate to form a virtual healthcare team able to carry out a healthcare workflow.

However, one of the major concern about the accomplishment of such a workflow regards how to guarantee the non-repudiation and immutability of all health decisions [6]. In recent years different solutions have been proposed to solve such an issue: among these, the Blockchain technology, thanks to its intrinsic features of data non-repudiation and immutability, has aroused a great interest in both scientific and industrial communities. One of the major applications of Blockchain regards smart contract, i.e., a computer protocol aimed to digitally facilitate, verify and enforce the negotiation of an agreement between subjects without the need of a certification third party. Blockchain has been increasingly recognized as a technology able to address existing information access problems in different applications domains including healthcare. In fact, it can potentially enhance the perception of safety around medical operators improving access to hospital Cloud services that are guaranteed by greater transparency, security, privacy, traceability and efficiency. Considering the tele-medical laboratory scenario, smart contracts can make the transactions related to the healthcare workflow track-able and irreversible.

Specifically, an architecture blueprint and a system prototype of FHC service enabling to address the healthcare workflow of a tele-medical laboratory scenario is proposed. In particular, a special emphasis is given to Blochchain comparing both public and hybrid (private/public) network scenarios using the Ethereum platform to assess both processing time and economic cost. In particular, the latter is necessary because the Ethereum public network platform available over the Internet requires that users (i.e., in our case federated Hospitals) pay a fee to perform each transaction.

The remainder of this paper is organized as follows. A brief overview of most recent initiatives about the adoption of Blockchain in healthcare is provided in Section 2. Motivations are discussed in Section 3. An blueprint of FHC architecture is presented in Section 4, whereas one of its possible implementations is described in Section 5. Experiments specifically focusing on Blockchain comparing public and hybrid network scenarios are discussed in Section 6. Section 7 concludes the paper also providing lights to the future.

## 2. Related Work

Recently, the role of Blockchain technology in the healthcare domain has been surveyed in several scientific works [7,8,9,10,11]. Blockchain can drastically improve the security of hospital information systems as discussed in [12,13,14,15]. However, up to now, most of the scientific initiatives are either theoretical or at an early stage and it is not always clear which protocols and pieces of a framework should be used to carry out system implementations that can be deployed in real healthcare environments.

Blockchain has been increasingly recognized as a tool able to address existing open information access issues [16]. In fact, it is possible to improve access to health services by using the Blockchain technology to achieve greater transparency, security and privacy, traceability and efficiency. In this regard, a solution adopting Blockchain with the purpose to guarantee authorized access to the patients’ medical information is discussed in [17]. In particular, mechanisms able to preserve both patient’s identity and the integrity of his/her clinical history is proposed. Another application of Blockchain regards the supply chain in the pharmaceutical sector and the development of measures against counterfeit drugs. While the development of new drugs involves substantial costs related to studies to evaluate the safety and updating of the drug, the use of smart contracts guarantees informed consent procedures and allows in certifying the quality of data [18]. An efficient data-sharing scheme, called MedChain is proposed in [19]. It combines Blockchain, digest chain and structured P2P network techniques to overcome the efficiency issues in healthcare data sharing. Experiments show that such a system can carry out higher efficiency and satisfy the security requirements of data sharing. As discussed in [20], different medical workflows have been designed and implemented using the Ethereum Blockchain platform which involves complex medical procedures like surgery and clinical trials. In particular, the smart contract system for healthcare management as been studied, also estimating associated costs in terms of feasibility. A piece of framework that integrates IoT networks with a Blockchain to address potential privacy and security risks for data integrity in healthcare is discussed in [21]. A Medical IoT Device represented by a Raspberry Pi 3 Model B+ is attached to the patient’s body to monitor his/her vital parameters that are stored in an Off-Chain Database that is accessed by doctor, pharmacy and insurance company via a DApp. All transactions take place utilizing smart contracts in a permissioned Blockchain system implemented employing Ethereum.

Differently from the aforementioned scientific initiatives, that are mainly based either on a public Blockchain networks approach, in this paper we focus on how a hybrid Blockchain network approach (mixing both private and public ones) can be used to carry out the healthcare workflow of a tele-medical laboratory running in a FHC environment. Moreover, we demonstrate that our Blockchain hybrid network approach allows reducing the number of required transactions, hence enhancing processing time and reducing economic cost.

## 3. Motivation

In this Section, after a brief overview of recent advances in medical laboratory devices, we discuss the advantages of tele-medical laboratory service.

### 3.1. Recent Advancements Medical Laboratory Devices

A medical laboratory device is an equipment able to perform several blood tests including CBC, Basic Metabolic Panel, Complete Metabolic Panel, Lipid Panel, Thyroid Panel, Enzyme Markers, Sexually Transmitted Disease Tests, Coagulation Panel and DHEA-Sulfate Serum Test. Currently, there are many medical laboratory devices available on the market. A classification can be done considering “connected” and “not connected” devices. For connected devices, we intend medical laboratory equipment including USB and network (wired and/or wireless) interfaces and able to export and send results to other devices, whereas for not connected devices, we intend medical laboratory devices without any interface for data transmission. In the following, we provide an overview of the major connected medical laboratory devices that are based on future tele-medical laboratory services. Telemedcare Clinical Monitoring Unit (CMU) [22] is a medical device able to perform blood pressure, pulse oximetry and blood glucose exams. Enverse [23] is a device able to perform continuous glucose monitoring. It consists of a chip that is installed subcutaneously on the patient that is connected with a mobile app. Med-Care [24] is an integrated solution for the auto-monitoring of glycemia that works with both web and mobile systems and that can send alerts via email or SMS. HemoScreen [25] is a low-cost portable haematology analyzer which performs a complete blood count at the point of care including a local web interface. Samsung Labgeo PT10S [26] is a portable clinical chemistry analyzer that improves efficiency by saving time for clinicians and patients through fast, easy and accurate blood analysis. It includes an ethernet interface for export exam result in an external Personal Computer (PC). All the aforementioned devices requires a blood sample in order to perform exams. Currently, alternative non-invasive experimental devices able to perform blood tests are the argument of study for both academic and industrial healthcare communities.

### 3.2. Towards Tele-Medical Laboratory

**Figure 1:**
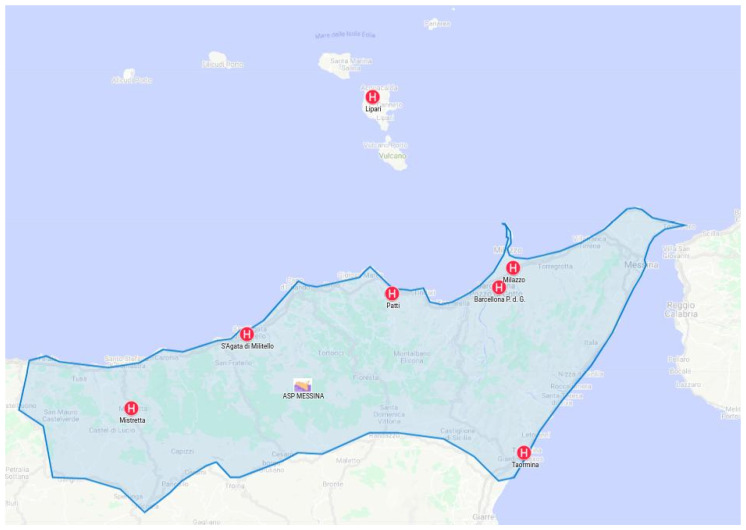
Federation of hospitals: clinical data is shared across participants for cooperation.

Tele-medical laboratory allows performing blood exams, results validation, diagnosis and therapy assignment tasks in departments located in different hospitals. This is possible utilizing the creation of a virtual healthcare team composed of biomedical laboratory health technicians and doctors belonging to different federated hospitals. Cooperation is possible through a federation of FHCs. Hospital federation can involve several satellite clinics belonging either to the same healthcare organization or to different ones. An example of healthcare organization including different hospital is the provincial healthcare organization of Messina (Italy), also referred to as ASP Messina. As shown in Figure 1, it includes eight health districts including, Messina, Taromina, Milazzo, Lipari, Barcellona Pozzo di Gotto, Mistretta and Sant’Agata di Militello. The health district of Lipari is placed on the island of Lipari and provides a limited number of health services. It offers a first aid to patients using an emergency room and a medical laboratory of clinical pathology. Due to the limited number of health departments, patients with particular diseases are typically transferred in the near health districts of Milazzo or Barcellona Pozzo di Gotto (indeed by helicopter for urgent cases) if required. In this scenario, a tele-medical laboratory service could help the accomplishment of a clinical workflow involving a virtual healthcare team including technicians and doctors belonging, for example to the Lipari, Milazzo and Barcellona Pozzo di Gotto districts. In particular, blood tests could be performed in the medical laboratory of Lipari by biomedical laboratory health technicians and results transmitted using the FHC ecosystem to a doctor of the Barcellona Pozzo di Gotto district for validations. Furthermore, leveraging the FHC environment an additional consultation could be done with a doctor of the Milazzo district.

Defining with the term “home hospital” the hospital that is physically reached by the patient, the generic healthcare workflow accomplishing the aforementioned scenario implies the following phases:

1. Hospitalization: patient reaches a home hospital; personal details, date and type of visit are recorded; the patient is identified by a visit identification code; a doctor schedules all exams required to verify the nature of the disease. A virtual healthcare team is created involving technicians, nurses and doctors belonging to the home hospital and other federated hospitals;
2. Clinical Analysis: if required, a nurse of the home hospital takes a blood sample from the patient; a biomedical laboratory health technician of the home hospital performs blood tests and results are saved by IoT medical devices automatically or by the technician manually on the FHC storage in a dedicated patient’s directory;
3. Validation: a doctor belonging to another federated hospital analyzes and validate the results of clinical analysis;
4. Consultation: a selected pool of doctors belonging to the virtual healthcare team establish a teleconference to clarify the patient’s clinical situation; the patient’s health data and clinical analysis are shared among doctors hiding sensitive data;
5. Monitoring: the hospitalized patient is constantly monitored by nurses who apply treatments based on therapeutic indications; each treatment is recorded until the dismissal.

Non-repudiation and immutability of all health decisions is a fundamental concern that must be addressed for the accomplishment of such a healthcare workflow. In this regard, the Blockchain technology using smart contracts can make all transactions related to the healthcare workflow track-able and irreversible. In the remainder of this paper, we will focus on such an aspect.

## 4. System Design

It is important to guarantee that only authorized members of the virtual healthcare team are allowed to take actions because a wrong decision can lead to a worsening of clinical condition or death of a patient.

**Figure 2:**
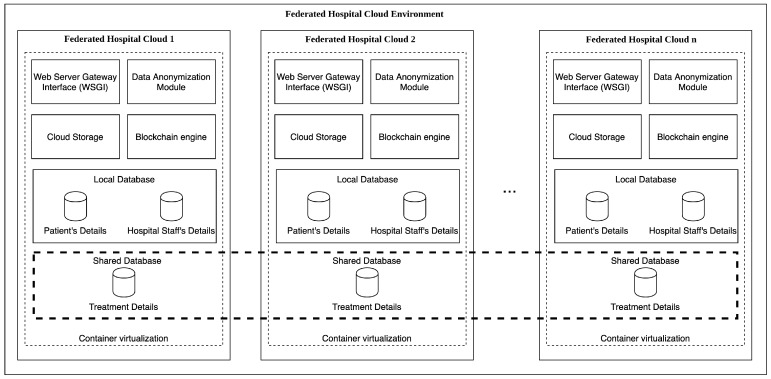
Federated Hospital Internet of Things (IoT) Cloud (FHC) architecture.

Therefore, the FHC system has to guarantee that all actions performed by virtual healthcare team members are track-able and irreversible. To achieve such a goal, the following technologies are fundamental:

- Blockchain engine: to use the features of a decentralized and distributed certification system with the technology offered by the development and coding of a smart contract.
- Cloud storage: to use an open-source and open-architecture file hosting service for file sharing managed with authorizations to archive all the files required to support the analysis of disease causes such as blood tests, Computed Tomography (CT) scans and laboratory tests;
- NoSQL database: to exploit the potential of a document-oriented distributed database able to store and manage patient’s data and diseases through tags for a fast and efficient search and to store Blockchain transaction hashes and links to files stored in Cloud Storage;

Figure 2 describes the FHC architecture. The system entry point of each hospital Cloud is a dedicated Web Server Gateway Interface (WSGI) where pieces of electronic healthcare record data are created manually by the clinical personnel or automatically collected by IoT devices that can be spread over different federated hospitals.

A Data Anonymization Module (DAM) is responsible for hiding the patient’s personal data in electronic health records. This is possible by decoupling the patient’s personal data from electronic health records storing related pieces of information in different databases. To bind the electronic health record with the patient, a patient’s anonymized identification number (patient_id) is generated from the DAM and stored within the electronic health record. Thus, only electronic health records are shared between FHCs, whereas pieces of patients’ personal data are never shared to preserve their privacy. All the produced clinical documentation, including electronic health records, is uploaded on a Cloud Storage containing a patient_id to hide patient’s data. A Blockchain engine is responsible to store information on the Blockchain to certify data non-repudiation and immutability of treatment details guaranteeing accountability and authenticity. The transaction hash resulting from the mining process is stored in the NoSQL document-oriented database as an attribute of the treatment. A local database instance containing both patients and hospital personnel data is isolated from other federated hospitals because these never require to be shared. Treatments’ details are anonymized and stored in a database shared with other participants in the FHC environment. The whole architecture deployed using container virtualization to simplify installation, configuration and maintenance in each FHC.

**Figure 3:**
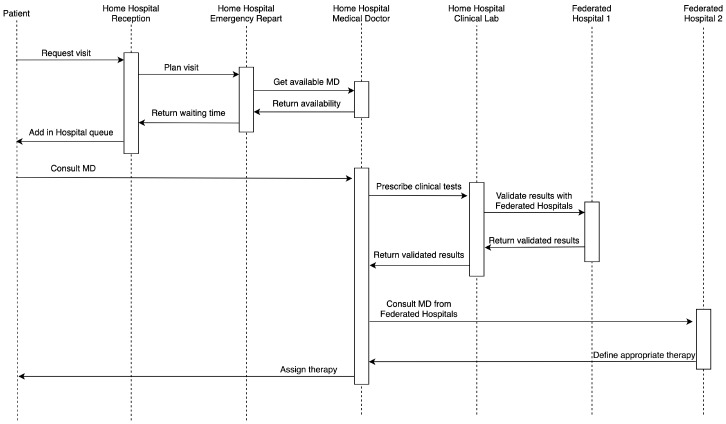
Sequence diagram describing an example of healthcare workflow accomplished in a Federated Cloud hospital environment.

Hospitals belonging to a Federation cooperate ensuring that appropriate therapies and procedures are carried out. Figure 3 shows the sequence diagram describing an example of healthcare workflow including three federated hospitals and where a tele-medical laboratory service is provided. A generic patient who requires a medical visit reaches the emergency room of a home hospital that after evaluating the urgency of the case, identifies an available doctor. At his/her turn, the patient is visited by the assigned doctor who prescribes some clinical analysis such as blood tests which can be done in the home hospital medical laboratory by a biomedical laboratory health technician. If the doctor responsible for the medical laboratory is not available (as the case of small hospital districts) a doctor belonging to the federated hospital (1) validates the results shared via federated Cloud storage. Thence, such a doctor joins the virtual healthcare team along with involved medical personnel of the home hospital. Since the doctor of the home hospital has a doubt the therapy to prescribe, he/she consults a doctor of federated hospital (2) via teleconference. After this consultation, a therapy is assigned to the patient.

## 5. System Prototype

**Figure 4:**
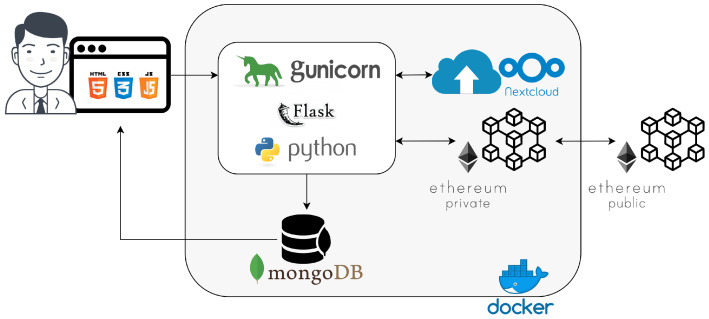
Hospital Cloud software components.

The FHC architecture was designed to enable a virtual healthcare team to carry out every healthcare workflow such as that described in the previous Section. Figure 4 shows the main software components of a possible system prototype implementation.

All requests coming from patients, nurses, technicians and doctors flow through the WSGI interface developed with the Python web application framework Flask and deployed on the Gunicorn Python WSGI HTTP server. All the components are configured as Docker containers to take the advantages of virtualization technology allowing service portability, resiliency and automatic updates that are typical of a Cloud Infrastructure as a Service (IaaS). The WSGI provides a front-end that allows retrieving all existing patients’ information (such as personal details, disease and pharmaceutic codes, links to clinical documentation and Blockchain hash verification); adding new patients; and submiting new treatments specifying all the required pieces of information. Specifically, a web page is dedicated to the registration of a new patient for saving his/her primary personal information and another web page is dedicated to the registration of a new treatment. It is possible to select the medical examination date, patient and doctor who does the registration.

All the produced clinical documentation is uploaded in a local instance of NextCloud storage using a folder for each treatment which does not contain any patient’s personal data, but a patient’s anonymized identification number. Every change in the files or content of the folder will be tracked making it possible to keep a history of the documentation and its modifications. Recently, a few related works were published for data anonymization in a Multi-Cloud storage environment considering a healthcare scenario, however, these do not guarantee that pieces of data are track-able and irreversible [27,28]. Since patients’ sensitive data must be anonymized and health records and treatments must be track-able and irreversible, related pieces of information were stored combining a MongoDB NoSQL DataBase Management System (DBMS) with the Ethereum Blockchain platform. Therefore, all pieces of information are stored in both MongoDB and in the Ethereum network through smart contracts developed in Solidity. For experimental purposes that will be discussed in Section 6, the Ethereum network was implemented in both public and hybrid configurations. In the first case, all FHCs share the public Ethereum network available over the Internet, whereas, in the second case each FHC hosts a private Ethereum network to store local transactions and a public Ethereum network to synchronize the local transactions performed in each FHC.

The smart contract accepts the input parameters such as anonymized patient id and doctor id, disease and pharmaceutic codes and stores these pieces of information in a simple data structure. The hash code resulting from the mining of each transaction is stored in the MongoDB database and can be used for verification using services like etherscan.io.

This service is capable of detecting any modification that occurred to files or a folder using a listener called External script. It is then possible to store the fingerprint and timestamp of each modification in the database thus making it possible to track the history of each treatment. This feature is important to guarantee the data integrity.

## 6. Experiments

Currently, the most adopted Blockchain configuration is based on a public network approach. With this regard, Ethereum is one of the major Blockchain platforms. A public Ethereum instance is available over the Internet and requires the payment of a fee for the execution of each transaction. However, the Ethereum platform can be also downloaded and installed in a private network. In this paper, using Ethereum, the objective of our experiments was to verify if the Blockchain system of a FHC ecosystem including a tele-medical laboratory service can be optimized in terms of both processing time and economic cost using the proposed hybrid network approach. Apart from processing time, it is important to highlight that considering the Ether (ETH) cryptocoin concurrency used in Ethereum, a small saving of ETH can result in putting aside a relevant amount of money (e.g., USD or EUR) in just a few months. In the following, we provide a description of both considered approaches.

- Ethereum public network: each healthcare treatment is recorded in the public Ethereum Blockchain network where time-to-mine and cost are subject to Ethereum network traffic (depending on the queue size, more time is required to extract transaction data, higher is the cost for transaction management).
- Ethereum hybrid network: each healthcare treatment is recorded in a private instance of Ethereum Blockchain network consisting of at least one node for each FHC and only one hash code, calculated as the MD5 of the last one-hundred concatenated treatments’ transaction hash, is written in the public Ethereum Blockchain network. In case the number of daily treatments is less than one-hundred, the MD5 hash code is calculated as the concatenation of the last 24 h’ treatments transactions hash. The result of this is a negligible waiting queue and ETH cost but, on the other hand, there is a reduction of the mining power as a reduced number of miners are present in the private network as compared to the public one.

The system assessment has been conducted analysing the total execution time required to perform a varying number of transactions in healthcare workflows. Each FHC was simulated considering a server with following hardware/software configuration: Intel^®^ Xeon^®^ E3-12xx v2 @ 2.7GHz, 4 core CPU, 4 GB RAM running Ubuntu Server 18.04. Each test has been repeated 30 times considering 95% confidence intervals and the average results are plotted.

Table 1 summarizes experiment setup and average outcomes.

**Figure 5:**
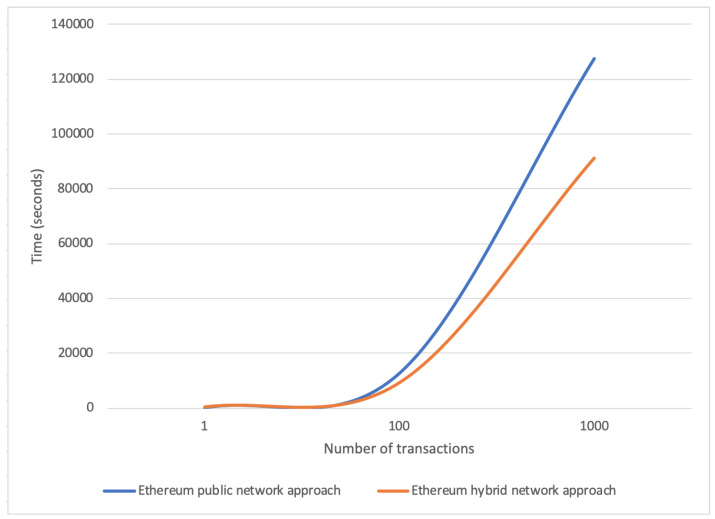
Time comparison for public and hybrid Ethereum network approaches considering a varying number of treatment registration requests.

Figure 5 shows the time-to-mine difference expressed in seconds between the two approaches considering new treatment registration requests. On the x-axis we reported the number of treatment registration requests, whereas on the y-axis we reported the processing time expressed in seconds. Looking at the graph, we can observe that for roughly 80 treatment registration requests both configurations present a similar trend, whereas increasing the number of requests the hybrid Ethereum network shows better performances than the public one thanks to the reduced waiting time in the mining queue.

**Figure 6:**
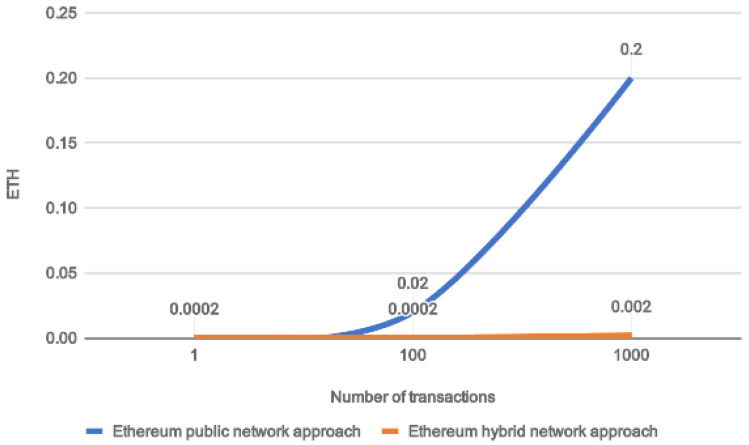
Cost comparison for public and hybrid Ethereum network approaches.

Figure 6 describes a cost comparison in Ether (ETH) for the two approaches. On the x-axis we reported the number of treatment registration requests, whereas on the y-axis we reported the cost expressed in Ether (ETH). From our tests, we appreciated that the average cost of a single transaction (i.e., a simple Smart Contract representing a new treatment memorization or modification) that has to be written in the public Ethereum Blockchain, is roughly 0.0002 ETH. It can be noted that for a small number of transactions there is not a perceptible convenience in preferring the proposed hybrid approach. This is because at least one transaction per day is written in the public Ethereum Blockchain. However, it is clear that the money-saving increases exponentially increasing the number of transactions because only one public transaction every one-hundred of private treatments will be paid with ETH cryptocurrency, resulting in an important cost-saving for the FHC ecosystem.

Test results demonstrate how the Ethereum hybrid network approach can be adopted to improve both processing time and cost-saving maintaining the same level of accountability and data certification as the public approach through certification on Blockchain.

## 7. Conclusions and Future Work

This paper demonstrated how a tele-medical laboratory service can be developed through a healthcare workflow running in a FHC environment leveraging Blockchain. Experimental results highlight that the performance of the Ethereum hybrid network certification system is improved in terms of cost and response time compared to an alternative public approach.

Definitely, the Blockchain technology is destined to evolve shortly improving system capabilities and robustness, and public test instances with different consensus protocols will be made available with benefits on performance and scalability.

In the pandemic condition that authors are living at the time of writing of this paper due to the Covid-19 virus, the need of tele-healthcare service becomes dramatically fundamental to reduce the infection risks for patients, thence reducing their movement. We hope that with this paper, we succeeded in stimulating the attention of both academic and industrial communities toward the adoption of Blockchain in the healthcare context to speed up the development of innovative tele-healthcare services.

In future developments, this work can be extended integrating a comprehensive healthcare scenario with different involved organizations, such as pharmaceutical companies registering in the Blockchain all the phases of drug production until the sealing of final package and shipment. Thus, when a patient buys a prescribed medicine it is possible to link the patient with the medicine box, which would mean an important step towards the end of drugs’ falsification and an important assurance for the end-user who can be identified in case a specific drug package has been recalled.

## Figures and Tables

**Table 1:** Summary of experiments performed.

Parameter	Value
Number of transactions tested	[1; 100; 1000]
Test executed for each run	30
Confidence interval	95%
Gas price (Gwei)	2
Average cost per transaction (ETH)	0.0002
Average mining time (s)	130

## References

[1] Hassenteufel P., Schweyer F.X., Gerlinger T., Henkel R., Lückenbach C., Reiter R. (2019). The role of professional groups in policy change: Physician’s organizations and the issue of local medical provision shortages in France and Germany. Eur. Policy Anal..

[2] Dubas-Jakóbczyk K., Domagała A., Mikos M. (2019). Impact of the doctor deficit on hospital management in Poland: A mixed-method study. Int. J. Health Plan. Manag..

[3] Moreno-Vozmediano R., Huedo E., Llorente I., Montero R., Massonet P., Villari M., Merlino G., Celesti A., Levin A., Schour L. (2016). BEACON: A cloud network federation framework. Commun. Comput. Inf. Sci..

[4] Celesti A., Fazio M., Giacobbe M., Puliafito A., Villari M. Characterizing cloud federation in IoT. Proceedings of the 2016 30th International Conference on Advanced Information Networking and Applications Workshops (WAINA).

[5] Mulfari D., Celesti A., Villari M., Puliafito A. How cloud computing can support on-demand assistive services. Proceedings of the 10th International Cross-Disciplinary Conference on Web Accessibility (ACM).

[6] Lian J.W., Yen D., Wang Y.T. (2014). An exploratory study to understand the critical factors affecting the decision to adopt cloud computing in Taiwan hospital. Int. J. Inf. Manag..

[7] Griggs K., Ossipova O., Kohlios C., Baccarini A., Howson E., Hayajneh T. (2018). Healthcare Blockchain System Using Smart Contracts for Secure Automated Remote Patient Monitoring. J. Med. Syst..

[8] Zubaydi H.D., Chong Y.W., Ko K., Hanshi S.M., Karuppayah S. (2019). A Review on the Role of Blockchain Technology in the Healthcare Domain. Electronics.

[9] Agbo C.C., Mahmoud Q.H., Eklund J.M. (2019). Blockchain Technology in Healthcare: A Systematic Review. Healthcare.

[10] Hölbl M., Kompara M., Kamišalić A., Nemec Zlatolas L. (2018). A Systematic Review of the Use of Blockchain in Healthcare. Symmetry.

[11] Qadri Y.A., Nauman A., Zikria Y.B., Vasilakos A.V., Kim S.W. (2020). The Future of Healthcare Internet of Things: A Survey of Emerging Technologies. IEEE Commun. Surv. Tutor..

[12] Chakraborty S., Aich S., Kim H. A Secure Healthcare System Design Framework using Blockchain Technology. Proceedings of the 2019 21st International Conference on Advanced Communication Technology (ICACT).

[13] Dasaklis T.K., Casino F., Patsakis C. Blockchain Meets Smart Health: Towards Next Generation Healthcare Services. Proceedings of the 2018 9th International Conference on Information, Intelligence, Systems and Applications (IISA).

[14] Srivastava G., Crichigno J., Dhar S. A Light and Secure Healthcare Blockchain for IoT Medical Devices. Proceedings of the 2019 IEEE Canadian Conference of Electrical and Computer Engineering (CCECE).

[15] Hossein K.M., Esmaeili M.E., Dargahi T., khonsari A. Blockchain-Based Privacy-Preserving Healthcare Architecture. Proceedings of the 2019 IEEE Canadian Conference of Electrical and Computer Engineering (CCECE).

[16] Zhang P., White J., Schmidt D., Lenz G., Rosenbloom S. (2018). FHIRChain: Applying Blockchain to Securely and Scalably Share Clinical Data. Comput. Struct. Biotechnol. J..

[17] Ramani V., Kumar T., Bracken A., Liyanage M., Ylianttila M. Secure and Efficient Data Accessibility in Blockchain Based Healthcare Systems. Proceedings of the 2018 IEEE Global Communications Conference (GLOBECOM).

[18] Biggs J., Hinish S.R., Natale M.A., Patronick M. (2017). Blockchain: Revolutionizing the Global Supply Chain by Building Trust and Transparency.

[19] Shen B., Guo J., Yang Y. (2019). MedChain: Efficient Healthcare Data Sharing via Blockchain. Appl. Sci..

[20] Khatoon A. (2020). A Blockchain-Based Smart Contract System for Healthcare Management. Electronics.

[21] Satamraju K.P. (2020). Proof of Concept of Scalable Integration of Internet of Things and Blockchain in Healthcare. Sensors.

[22] Telemedcare Clinical Monitoring Unit (CMU). https://www.telemedcare.com/.

[23] Enverse Continous Glucose Monitoring. https://www.eversensediabetes.com/.

[24] Med-Care. Http://www.t4all.it/portfolio-articoli/med-care/.

[25] HomoScreen. https://www.pixcell-medical.com/products/hemoscreen.

[26] Samsung Labgeo PT10S. https://samsunghealthcare.com/en/products/InVitroDiagnostics/Samsung%20LABGEO%20PT10/Point-of-Care/benefit.

[27] Galletta A., Bonanno L., Celesti A., Marino S., Bramanti P., Villari M. An approach to share MRI data over the Cloud preserving patients’ privacy. Proceedings of the 2017 IEEE Symposium on Computers and Communications (ISCC).

[28] Galletta A., Celesti A., Tusa F., Fazio M., Bramanti P., Villari M. (2017). Big MRI Data Dissemination and Retrieval in a Multi-Cloud Hospital Storage System. Proceedings of the 2017 International Conference on Digital Health.

